# Determinants of acute kidney injury in children with new onset type 1 diabetes: A cohort study of children aged <15 years: Auckland, New Zealand (2006–2016)

**DOI:** 10.1002/edm2.362

**Published:** 2022-08-04

**Authors:** Fiona Pittman, Harry Di Somma, William Wong, Chanel Prestidge, Peter Reed, Alistair J. Gunn, Craig Jefferies

**Affiliations:** ^1^ Starship Children's Health Auckland New Zealand; ^2^ Auckland Medical School University of Auckland Auckland New Zealand; ^3^ Department of Physiology Auckland Medical School, University of Auckland Auckland New Zealand; ^4^ Liggins institute University of Auckland Auckland New Zealand; ^5^ Department of Paediatrics University of Auckland Auckland New Zealand

**Keywords:** diabetes, ketoacidosis, kidney

## Abstract

**Objective:**

Acute kidney injury (AKI) may contribute to the risk of diabetic kidney disease, however, there have been limited studies of the incidence of AKI in well‐defined populations of children with type 1 diabetes. The aim was to quantify AKI in children presenting with new onset type 1 diabetes from the regional paediatric diabetes service, Auckland, New Zealand.

**Research Design and Methods:**

A retrospective analysis of a prospectively identified cohort study of children and adolescents presenting from 2006 to 2016 with type 1 diabetes aged <15 years. AKI was defined using Kidney Disease/Improving Global Outcomes serum creatinine criteria.

**Results:**

There were 586 subjects: 52% male, with mean (*SD*) age 8.9 (3.8) years, with 151(25.8%) in diabetic ketoacidosis (DKA). AKI was present in 47%, 278/586, AKI was increased in those with DKA (125/151 (83%) DKA vs. 153/435 (35%) no‐DKA). Univariable analysis showed that increased HbA1c, higher glucose levels, lower BMI SDS, lower bicarbonate and pH levels were all associated with AKI (*p* < .001). In multivariable analysis, AKI was associated with DKA and higher glucose levels independently. The majority of cases were stage 1 (203/278 [73%]), or stage 2 AKI 62/278 (22%). 13/278 (5%) had severe, Stage 3 AKI, and all presented in DKA (13/151 (8%) vs. 0/435 (0%) without DKA, *p* < .001).

**Conclusion:**

In this regional paediatric, cohort AKI is a common complication of children presenting with new onset type 1 diabetes. AKI is independently associated with higher glucose levels and DKA, and all cases of Stage 3 AKI were associated with DKA.

## Abstract 2

In this regional paediatric cohort AKI is a common complication of children presenting with new onset type 1 diabetes. AKI is independently associated with higher glucose levels and DKA, and all cases of Stage 3 AKI were associated with DKA.

**What is already known?**

- Acute kidney injury is increased in children presenting with DKA from select studies in intensive care settings.

**What does this study add?**

- This study confirms the association with DKA but that hyperglycaemia is also a risk factor without DKA in a regional cohort.

**How might these results impact pre‐clinical or clinical perspectives or research?**

- Increasing awareness of the both the rates of AKI in new onset and the association with DKA is an area that if recognized can be more aggressively treated, furthermore it adds more weight to recognizing diabetes as a risk factor to preventing acute kidney damage.

## 1 INTRODUCTION

Type 1 diabetes mellitus is one of the most common chronic diseases amongst children and adolescents and is increasing worldwide.^1^ Although diabetes‐related severe chronic kidney injury is rare in children and adolescents, there is increasing evidence that it may be associated with unrecognized acute kidney injury (AKI), particularly after diabetic ketoacidosis (DKA).^2^

Hursh et al.^3^ highlighted in a cohort of 165 children and adolescents in DKA from a tertiary unit that 64.2% developed AKI, including 21/106 (19.8%) with severe (stage 3) AKI, of whom two required haemodialysis. AKI was associated with lower serum HCO3 levels (<10 mmol/L) and evidence of more severe volume depletion. A similar percentage of AKI in DKA has been shown by Huang^4^ with 170 (56.5%) patients with DKA presented in AKI. Subsequently the DKA Fluid Study Group showed that 43% presenting in AKI (43%) in a larger prospective cohort of children presenting with DKA (47.9% with new onset DKA) and highlighted the association with greater acidosis and circulatory volume depletion.^5^ All these studies were done in children presenting with DKA or from tertiary referral intensive care units. There is evidence in adults now that diabetes is associated with greater risk of AKI and long‐term mortality/morbidity.^6^

AKI represents an abrupt reduction in kidney function, resulting in retention of urea, other waste products and dysregulation of extracellular volume and electrolyte homeostasis.^2^ The association of AKI with DKA is presumptively related to progressive extracellular volume depletion with prerenal failure that ultimately leads to renal hypoperfusion and acute tubular injury.^7^ There is growing evidence that AKI may contribute to later chronic kidney disease in the non‐diabetic population; for example Greenberg et al. (2014) found in 346 children followed for a mean of 6.5 years after a single episode of AKI that proteinuria developed in 3.1%, hypertension in 1.4%, GFR <60 ml.min/1.73 m^2^ in 6.3% and end‐stage renal disease in 0.8%. This suggests that even a single episode of AKI in children may be associated with an elevated risk of long‐term renal outcomes. However, the causal relationship between AKI and CKD needs more in‐depth study.^8^

New Zealand has a long‐established social security system that provides free medical care (private insurance is not required) for all citizens and permanent residents. All cases of new onset type 1 diabetes in Auckland <15 years of age are initially managed in a publicly funded tertiary institution, Starship Children's Health, a regional paediatric diabetes centre for the Auckland Metropolitan area. This allows population‐based assessment of factors associated with type 1 diabetes.

In this study, we aimed to determine the incidence and severity of AKI in this regional population of children/adolescents aged less than 15 years presenting with new onset type 1 diabetes for the years 2006 to 2016 inclusive. We then looked to evaluate the potential determinants of AKI such as diabetic ketoacidosis (DKA), hyperglycaemia and acidosis, and whether the risk of AKI was associated with age, sex, socioeconomic status or ethnicity.

## 2 METHODS

### 2.1 Data source

This study was undertaken at The Starship Children's Hospital in Auckland, New Zealand, the largest Paediatric hospital in New Zealand, and the only service providing diabetes care in the Auckland region. All children and adolescents presenting with type 1 diabetes who were less than 15 years of age and had presented between 1st January 2006 and 31st December 2016 were included in the study. Data sources included all new onset cases of type 1 diabetes from our local diabetes database, comparison to intensive care and renal databases for renal replacement therapy and with hospital‐based medical records. For each patient included in the study, type 1 diabetes was diagnosed according to the 2018 ISPAD guidelines.^9^ All patients had elevated blood glucose at presentation (a random blood glucose of >11.1 mmol/L and/or fasting blood glucose >7.1 mmol/L) and presented with classical symptoms. In addition, all patients met at least one of the following criteria: (a) diabetic ketoacidosis; (b) presence of pre‐type 1 diabetes‐associated antibodies (glutamic acid decarboxylase, islet antigen 2, islet cell or insulin autoantibodies); or (c) on‐going requirement for insulin therapy, as per our previous publications.^10^, ^11^ DKA was defined according to ISPAD 2018 guidelines^12^ and classified as mild (pH < 7.3 or HCO3 < 15 mmol/L), moderate (pH < 7.2 or HCO3 < 10 mmol/L) or severe (pH < 7.1 or HCO3 < 5 mmol/L).

### 2.2 Data collection

The following data were collated on all eligible patients from our diabetes database: age of type 1 diabetes onset, ethnicity, gender, height and weight at diagnosis, glucose, and hbA1c. Additional data for the purposes of this study on renal function indices included creatinine and information on renal replacement therapy. During the study period, 2006–2016, all creatinine levels were measured using the modified Jaffe method (Roche Diagnostic) (from 2017 on an enzymatic assay was introduced the centralized lab, hence we restricted our analysis to the period prior 2017). Demographic data were collected at diagnosis. Ethnicity was recorded throughout the study period by self‐report using a standardized prioritized system as follows: if multiple ethnicities were selected, the patient was assigned to a single ethnicity by an established hierarchical classification of Māori, Pacific Islander (Pasifika), Other and then New Zealand European.^13^ ‘Other’ included Indian, Asian, African and Middle Eastern ethnicities, with the majority of Other being Asian.^10^ Socioeconomic status was determined using the New Zealand Index of Deprivation 2013 (NZDep2013), a geo‐coded deprivation score derived from current residential address, reflecting nine aspects of material and social deprivation to divide New Zealand into tenths (scored 1–10).^14^

The data that support the findings of this study are available on request from the corresponding author. The data are not publicly available due to privacy or ethical restrictions.

### 2.3 Case definitions

The primary outcome variable, AKI, was defined by the Kidney Disease/Improving Global Outcomes (KDIGO) serum creatinine criteria.^7^ Because no study participants had available baseline serum creatinine values prior to admission, we used an estimated glomerular filtration rate (eGFR) of 120 ml/min/1.73 m^2^ to calculate an expected baseline creatinine level (EBC) using the Schwartz estimating Equation.^15^ An eGFR of 120 ml/min/1.73 m^2^ was selected for consistency with a number of previous published studies of paediatric AKI.^3^, ^4^, ^5^ The estimated GFR (eGFR) calculated from the derived serum creatinine is considered a conservative estimate as normal GFR ranges from 90 to 130 ml/min/1.73 m^2^, Llanos‐Paez^16^ considered that this was the most appropriate measure of eGFR.

AKI was defined as follows: an increase in serum creatinine by ≥26.5 μmol/L within 48 hours; or an increase in serum creatinine to greater than 1.5 times estimated baseline, which is known or presumed to have occurred within the prior 7 days.^17^, ^18^ The severity of AKI was stratified as follows: Stage‐1 AKI serum creatinine 1.5 to less than 2 times higher than baseline creatinine, Stage‐2 AKI serum creatinine 2 to <3 times higher than the baseline creatinine and Stage‐3 AKI if serum creatinine was 3 or more times higher than the estimated baseline creatinine.^17^, ^18^

BMI‐standard deviation scores (SDS) are based on the 2000 CDC growth charts and were calculated using the LMSgrowth MS‐excel add‐in (Harlow Healthcare, South Shields, UK).

Ethics approval was granted by the Auckland District Health Board Research Review Committee (study number A+ 7654). All procedures followed were in accordance with the ethical standards of the responsible committees.

### 2.4 Subjects

There were 697 eligible children with newly diagnosed type 1 diabetes between January 2006 and December 2016 identified from the diabetes database. On review of all eligible cases, the following were excluded: 104 who had minimal biochemistry records and no serum creatinine measured and or was not admitted, (all this group were not in DKA and hence had minimal tests), diagnosis was made whilst overseas (*n* = 1), earlier diagnosis of type 1 diabetes (*n* = 2), re‐classified as type 2 diabetes or monogenic diabetes (*n* = 4). The final study population was therefore 586.

### 2.5 Statistical analysis

Data were analysed using JMP ver15.0 (SAS Inc.). Continuous measures were compared using ANOVA, categorical measures were compared using Fisher's exact test. A *p* value of <.05 was considered significant. The independence of factors associated with AKI was examined by multivariable logistic regression.

## 3 RESULTS

There were 586 cases of new onset type 1 diabetes with an average age (*SD*) of 8.93 (3.8) years (interquartile range 0.7–16.0) years. 51.9% were male (Tables 1 and 2). AKI was present in 278/586 (47% of cases), the majority 203/586 (35%) had stage 1 AKI, 62/586 (11%) stage 2 AKI and 13/586 (2%) stage 3 AKI, of whom two required renal replacement treatment.

**TABLE 1:** Demographics of all subjects and according to AKI status

	All subjects	AKI	No AKI	*p* Value*
Demographics	586	278/586 (47%)	308/586 (53%)	
Age in years (*SD*)	8.9 (3.8)	8.7 (4.0)	9.1 (3.6)	.24
Sex (M)	304 (52%)	149/304 (49%)	155/304 (51%)	.46
Ethnicity				
Māori	77/586 (13%)	35/77 (45%)	42/77 (55%)	.12
Pasifika	69/586 (12%)	42/69 (61%)	27/69 (39%)	
Other	75/586 (13%)	36/75 (48%)	39/75 (52%)	
European	365/586 (62%)	165/365 (45%)	200/365 (55%)	
NZ Dep score (*SD*)	4.8 (2.9)	5.0 (3.0)	4.6 (2.9)	.15
Anthropometry (*SD*)				
Height (cm)	136 (25)	134 (26)	137 (23)	.07
Height SDS	0.68 (1.0)	0.57 (1.1)	0.77 (1.0)	.02
Weight (kg)	33.5 (16.2)	31.5 (15.3)	35.2 (16.9)	.005
Weight SDS	0.21 (1.1)	−0.01 (1.1)	0.40 (1.1)	<.0001
BMI (kg/m^2^)	17.1 (3.5)	16.6 (3.1)	17.6 (3.8)	.0003
BMI SDS	−0.25 (1.4)	−0.52 (1.4)	−0.01 (1.3)	<.0001

* Data were analyzed using JMP ver15.0 (SAS Inc.). Continuous measures were compared using ANOVA, categorical measures were compared using Fisher's exact test, *p* value of <.05 considered significant.

**TABLE 2:** Biochemical parameters of all subjects and by AKI status

	All subjects	AKI	No AKI	Significance
Parameters (*SD*)	586	278/586 (47%)	308/586 (53%)	
Glucose (mmol)	27.1 (10.2)	31.4 (10.5)	23.1 (8.2)	<.0001
HbA1c (mmol/mol)	108.8 (28)	114.7 (26)	103.7 (30)	<.0001
HbA1c (%)	12.1 (2.6)	12.6 (2.3)	11.6 (2.7)	
pH	7.3 (0.1)	7.3 (0.1)	7.4 (0.1)	<.0001
Bicarbonate (mmol/l)	19.2 (5.9)	16.5 (6.4)	21.7 (4.0)	<.0001
DKA	151/586 (26%)	125/278 (45%)	26/308 (8%)	<.0001
DKA mild	70/586 (12%)	57/70 (81%)	13/70 (19%)	
DKA mod	42/586 (7%)	31/42 (74%)	11/42 (26%)	
DKA severe	39/586 (7%)	37/39 (95%)	2/39 (5%)	
Renal indices (*SD*)				
Creatinine (μmol/l)	62 (23)	75 (26)	51 (11)	<.0001
eGFR (ml/min/1.73 m^2^)	85 (22)	67 (13)	100 (15)	<.0001
AKI stage			–	<.001
Stage 1 AKI	203/586 (35%)	203/278 (73%)	–	–
Stage 2 AKI	62/586 (11%)	62/278 (22%)	–	–
Stage 3 AKI	13/586 (2%)	13/278 (5%)	–	–

There were no statistical differences in AKI by age, ethnicity, or deprivation indices. On univariate analysis, AKI was associated with measures of weight loss and poor growth: lower BMI SDS, lower weight SDS and lower height SDS. Biochemically, AKI was associated with higher glucose and higher HbA1c and with lower pH and bicarbonate (Tables 1 and 2). In multivariable logistic regression, AKI was independently associated with the presence of DKA (*p* < .0001, odds ratio 8.99, 95%CI 5.41–14.9) and higher glucose levels (*p* < .0001, odds ratio per mmol, 1.12, 95%CI 1.09–1.15).

The percentage of AKI was higher in those children presenting in DKA 125/151 (83%) than the percentage of AKI in the children not presenting in DKA, 153/435 (35%) *p* < .0001, see Tables 3 and 4. There were neither statistical differences in age, % male, or ethnicity nor deprivation. Height SDS was not significant, but weight SDS and BMI SDS were lower in subjects with DKA. Glucose parameters were increased with DKA (higher glucose and HbA1c). The risk of moderate to severe AKI was greater in children with DKA: stage 2 AKI (42/151 (28%) vs. 20/435 (5%)) and stage 3 AKI 12/151 (8%) vs. 0/435 (0%), respectively.

**TABLE 3:** Demographics according to presence or absence of DKA

	All subjects	DKA	No DKA	Significance
Demographics	586	151 (26%)	435 (74%)	
Age in years (*SD*)	8.9 (3.8)	9.3 (3.9)	8.8 (3.8)	.15
Sex (M)	304 (52%)	68 (22%)	236 (78%)	.06
Ethnicity				.06
Māori	77/586 (13%)	14/77 (18%)	63/77 (82%)	–
Pasifika	69/586 (12%)	25/69 (36%)	44/69 (64%)	–
Other	75/586 (13%)	23/75 (31%)	52/75 (69%)	–
European	365/586 (62%)	89/365 (24%)	276/365 (76%)	
Anthropometric measures				
NZ Dep score	4.8 (2.9)	5.1 (3.0)	4.7 (2.9)	.11
Height (cm)	136 (25)	137 (25)	135 (25)	.54
Height SDS	0.68 (1.0)	0.54 (1.0)	0.73 (1.1)	.06
Weight (kg)	33.5 (16.2)	31.8 (13.6)	34.0 (17.0)	.16
Weight SDS	0.21 (1.1)	−0.25 (1.1)	0.37 (1.1)	<.0001
BMI (kg/m^2^)	17.1 (3.5)	16.2 (2.9)	17.4 (3.6)	.0001
BMI SDS	−0.25 (1.4)	−0.85 (1.5)	−0.05 (1.3)	<.0001
BSA (m^2^)	1.11 (0.4)	1.09 (0.3)	1.12 (0.4)	.42

**TABLE 4:** Biochemical parameters by presence or absence of DKA

	All subjects	DKA	No DKA	Significance
*N* (%)	586	151 (26%)	435 (74%)	
Glucose (mmol)	27.1 (10.2)	30.5 (12.0)	25.9 (9.2)	<.0001
HbA1c (mmol/mol)	108.8 (28)	117.3 (23)	106.3 (29)	.0001
HbA1c (%)	12.1 (2.6)	12.9 (2.1)	11.9 (2.7)	
pH	7.3 (0.1)	7.2 (0.1)	7.4 (0.1)	<.0001
Bicarbonate (mmol/L)	19.2 (5.9)	10.7 (3.7)	22.2 (2.7)	<.0001
DKA	151/586 (26%)		**–**	**–**
DKA mild		70/151 (46%)	–	–
DKA mod		42/151 (28%)	–	–
DKA severe		39/151 (26%)	–	–
Renal indices				
Creatinine (μmol/l)		79 (31)	56(16)	<.0001
eGFR (ml/min/1.73 m^2^)		68 (19)	90 (19)	<.0001
AKI		125/151 (83%)	153/435 (35%)	<.0001
Stage 1 AKI		71/151 (47%)	132/435 (30%)	
Stage 2 AKI		42/151 (28%)	20/435 (5%)	
Stage 3 AKI		12/151 (8%)	1/435 (0%)	

## 4 DISCUSSION

This study in an unselected regional population confirms that AKI is common in all children presenting with new onset type 1 diabetes, and also highlights the strong association with DKA. Furthermore, it confirms that DKA is highly associated with both the risk and severity of AKI. Overall, we have shown that DKA and higher glucose levels were both independently associated with AKI. A considerable number had moderate to severe AKI (stage 2 or 3). This implies that these cases are likely to have also developed intrinsic tubular injury, a finding confirmed by several recent studies in children in DKA.^3^, ^19^, ^20^

There are few if any studies in the paediatric literature to compare rates of AKI in common paediatric conditions, such as gastroenteritis. The 3 most common causes of hospital acquired AKI are renal perfusion insufficiency, nephrotoxic drugs and contrast agents, two of these are at least potentially avoidable and not seen in this ‘pre‐hospital’ study.^21^ In this study, chart review showed that <5% had received oral antibiotics and there were no known cases of NSAID use. Comparatively a small number (<5%) in the DKA group received a 10–20 ml/kg bolus of fluid prior to arrival in hospital at primary care or ambulance. We did not evaluate this sub‐group as the number was too small. However, the effect on creatinine values would have been small and would have tended to reduce. From a local point of view, we were unable to show any difference in AKI due to ethnicity or deprivation indices, previous work by our group shows that metabolic control differs along these lines markedly.^13^

Studies in adults with diabetes also suggest that diabetes is associated with AKI.^6^ Girman reported that the annual AKI incidence in a General Practice Research Database from the United Kingdom of type 2 diabetes was 198/100,000 and 27/100,000 subjects, respectively.^22^ Comparatively, Oliveira et al. found a prevalence of DM of 19.6 versus 9.3% in AKI compared to no‐AKI in their prospective analysis.^23^

One limitation of this and other studies of AKI is the reliance on estimated baseline GFR to assess changes in renal function.^3^, ^4^ However similar rates of AKI in a DKA population using the stable creatinine in the recovery phase suggest this estimate it not invalid.^20^ Interestingly if one considers that the presentation of new onset type 1 diabetes has in most cases been heralded by marked weight loss (and muscle loss) and days if not weeks of hyperglycaemia, how ‘acute’ the AKI is not known. Our finding of increased AKI with markers of weight loss (lower BMI SDS) and associated higher creatinine levels suggest that we may be underestimating the renal function abnormalities relying on creatinine values. In the recently published prospective study by Marzuillo et al. (2021), they demonstrated not just creatinine abnormalities, but increased markers of renal tubular dysfunction, tests that were not available to us in this study.^20^

The impact of AKI on long‐term risk of renal impairment is still unclear. Recent studies and data suggest that episodes of AKI are not benign, and the more severe the episode of AKI the increased risk of chronic kidney disease.^24^ Recognition of AKI is an important first line to consider; in our study, 46.7% of patients who were diagnosed with stage 1 AKI did not have repeated creatinine measurements prior to discharge. It is therefore important to assess the serum creatinine and ensure proper systems and guidelines are in place to follow‐up on those with abnormal levels. Prospective longitudinal studies are therefore needed to establish the likelihood of renal dysfunction in children with diabetes, as well as the best treatment options once clinicians are more aware of AKI.^20^

### 4.1 Strengths and limitations

The strength of this study is that all children with new onset type 1 diabetes in the Auckland region are diagnosed and managed in a single centre with prospective case recording. The main limitation in this study is the absence of a baseline serum creatinine level for the children. It is reasonable to consider that the Schwartz equation was designed to measure renal function in children with chronic kidney disease with relatively stable creatinine levels as opposed to the dynamic changes in many of our cases.^15^ In using this equation in this way, it was estimated that a baseline GFR of 120 ml/min/1.73 m^2^ was most appropriate, and in line with most publications to date; however the use of this estimation is problematic, as the initial publication by Schwartz was in children with established type 1 diabetes and the upper limit of 120 is not valid in all age groups of children.^15^ Finally, this was a retrospective study, and a although all subjects with DKA had creatinine's performed, there were a number of well children not in DKA who did not have available creatinine levels. Nearly all of these patients were identified from type 1 diabetes screening and were asymptomatic, however it does mean that our rate of AKI in our non‐DKA group may have a degree of overestimate bias, (that the patients who were well did not get creatinine levels and were therefore excluded from this study).

## 5 CONCLUSIONS

This study shows that mild to moderate AKI is common in children presenting with new onset type 1 diabetes with or without DKA. However, AKI was independently associated with both DKA and hyperglycaemia, and severe AKI was only present in children who were in DKA. Further research on the long‐term impact of AKI on later risk of diabetic renal disease and metabolic control will be important.

## AUTHOR CONTRIBUTIONS

**Fiona Pittman:** Data curation (equal); methodology (equal); writing – original draft (equal). **Harry Di Soma:** Data curation (equal); methodology (equal); writing – original draft (equal). **William Wong:** Conceptualization (equal); methodology (equal); writing – original draft (equal); writing – review and editing (equal). **Chanel Prestidge:** Conceptualization (equal); methodology (equal); writing – review and editing (equal). **Peter Reed:** Methodology (equal); project administration (equal); software (lead); writing – original draft (equal). **Alistair J. Gunn:** Methodology (equal); validation (equal); writing – original draft (equal); writing – review and editing (equal). **Craig Jefferies:** Conceptualization (lead); funding acquisition (equal); methodology (equal); project administration (equal); resources (equal); writing – original draft (lead); writing – review and editing (lead).

## CONFLICTS OF INTEREST

The authors have no financial or non‐financial conflicts of interest to disclose.

## Data Availability

The data that support the findings of this study are available on request from the corresponding author. The data are not publicly available due to privacy or ethical restrictions.
